# Structured line illumination Raman microscopy

**DOI:** 10.1038/ncomms10095

**Published:** 2015-12-02

**Authors:** Kozue Watanabe, Almar F. Palonpon, Nicholas I. Smith, Liang-da Chiu, Atsushi Kasai, Hitoshi Hashimoto, Satoshi Kawata, Katsumasa Fujita

**Affiliations:** 1 Department of Applied Physics, Graduate School of Engineering, Osaka University, 2-1 Yamadaoka, Suita, Osaka 565-0871, Japan; 2 Immunology Frontier Research Center, Osaka University, 3-1 Yamadaoka, Suita, Osaka 565-0871, Japan; 3 Laboratory of Molecular Neuropharmacology, Graduate School of Pharmaceutical Sciences, Osaka University, 1-6 Yamadaoka, Suita, Osaka 565-0871, Japan; 4 Interdisciplinary Program for Biomedical Sciences, Institute for Academic Initiatives, Osaka University, 1-6 Yamadaoka, Suita, Osaka 565-0871, Japan; 5 iPS Cell-based Research Project on Brain Neuropharmacology and Toxicology, Graduate school of Pharmaceutical Sciences, Osaka University, 1-6 Yamadaoka, Suita, Osaka 565-0871, Japan; 6 Molecular Research Center for Children's Mental Development, United Graduate School of Child Development, Osaka University, Kanazawa University, Hamamatsu University School of Medicine, Chiba University and University of Fukui, 2-2 Yamadaoka, Suita, Osaka 565-0871, Japan

## Abstract

In the last couple of decades, the spatial resolution in optical microscopy has increased to unprecedented levels by exploiting the fluorescence properties of the probe. At about the same time, Raman imaging techniques have emerged as a way to image inherent chemical information in a sample without using fluorescent probes. However, in many applications, the achievable resolution is limited to about half the wavelength of excitation light. Here we report the use of structured illumination to increase the spatial resolution of label-free spontaneous Raman microscopy, generating highly detailed spatial contrast from the ensemble of molecular information in the sample. Using structured line illumination in slit-scanning Raman microscopy, we demonstrate a marked improvement in spatial resolution and show the applicability to a range of samples, including both biological and inorganic chemical component mapping. This technique is expected to contribute towards greater understanding of chemical component distributions in organic and inorganic materials.

## Abstract (web summary)

Raman imaging provides visualization of molecular content in a sample, but has been practically limited in resolution. Here, the authors implement structured line illumination in slit-scanning Raman microscopy, expanding its resolvable spatial frequencies without spoiling the advantage of the Raman approach.

## Graphical abstract

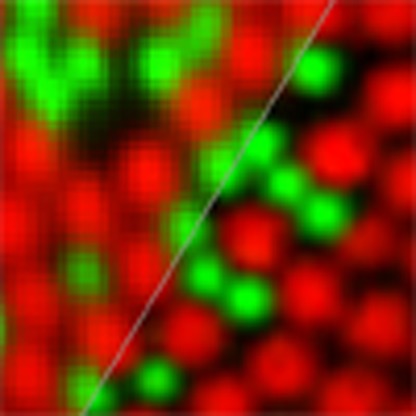

The approaches toward improvement of the spatial resolution in optical microscopy have been successful in the last couple of decades. In these achievements, the excitation and emission properties of fluorescence probes play a key role to overcome the classical spatial resolution limitation^1^. Localization microscopy achieves a few tens of nanometre resolution by localizing single fluorescent molecules with stochastic emission^2^,^3^,^4^. Stimulated emission depletion microscopy and equivalent techniques, such as reversible saturable optical fluorescence transitions microscopy, significantly decrease the detection volume by depleting fluorescence emission through stimulated emission or saturated transition of fluorescence probes to an undetectable state^5^,^6^,^7^. Structured illumination microscopy (SIM) illuminates a sample by using an illumination with a grid pattern producing the moiré effect to make small structure information transferable through the imaging optics, resulting in a doubling of the spatial resolution compared with the classical limit^8^,^9^. These approaches are usually grounded in modification of a fluorescence-based imaging method. Although a few techniques have been demonstrated for super-resolution imaging of non-fluorescent samples^10^,^11^, there has been much less progress in achieving similar super resolution gains in far-field spontaneous Raman microscopy.

Among the different super-resolution techniques, the structured illumination method is an ideal first choice for spatial resolution improvement in Raman microscopy as it does not impose particular optical properties, such as switching, on the choice of samples. As the main applications of Raman microscopy lie within its capability of materials analysis, many applications preclude modification of the sample. The structured illumination in essence overlays a fine illumination pattern on a sample to expand spatial frequencies resolvable by the optics without spoiling the analytical advantage of Raman microscopy.

In this report, we implemented structured line illumination in slit-scanning Raman microscopy where a line-shaped focus illuminates the sample and scanning in one axis produces a two-dimensional (2D) Raman image^12^,^13^,^14^. The line illumination lends itself easily to application of a structured pattern along the direction parallel to the line, where the spatial resolution is expected to be enhanced. For Raman microscopy, the spectrum measurement is key, and is the basis of its importance to various Raman imaging applications. High spectral resolution and relatively high imaging speed are maintained in structured-line-illumination (SLI) Raman microscopy because of the parallel detection of Raman scattering through the spectrophotometer slit. Without both of these features, the possible applications are restricted. An analogous configuration has been applied to fluorescence microscopy to improve the imaging capability in thick specimens via a slit confocal detection that strongly reduces the out-of-focus signals^15^,^16^. This benefit is also granted to SLI Raman microscopy.

## Results

### SLI Raman microscopy

**Figure 1:**
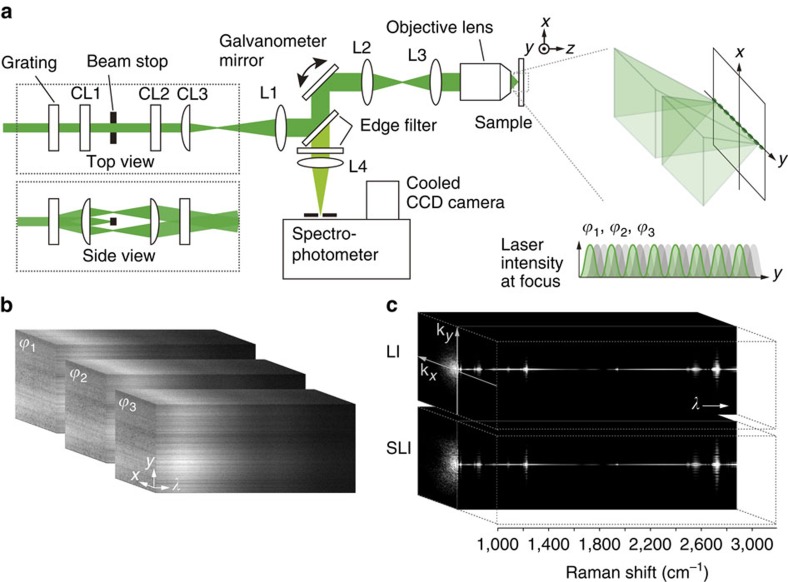
SLI Raman microscopy. (**a**) A schematic optical system of SLI Raman microscopy. CL and L represent a cylindrical lens and lens, respectively. (**b**) Hyperspectral images of a fluorescent PMMA film of Rhodamine 6G obtained with three different fringe phases. The effect of structured illumination is distinctly seen in the entire wavelength detection range. (**c**) The spatial frequencies of line illumination (LI) and structured line illumination (SLI) Raman images of polystyrene beads with a diameter of 330 nm. The improvement of the spatial resolution can be confirmed in all the detected Raman bands from the sample.

Figure 1a shows an optical system for SLI Raman microscopy. Light from the laser is split by a phase grating to produce interference fringes in the line illumination at the sample. Raman scattering from the sample produces a projection of the line on the spectrophotometer slit and is then detected as a one-dimensional (1D) hyperspectral image on a cooled CCD camera (Fig. 1b). We obtained 1D hyperspectral images with three different fringe phases for image reconstruction. The fringe phase was varied by moving the position of the grating using a piezo scanner. After taking three 1D images at one position, the SLI line focus was scanned to the adjacent position to step-wise obtain a full 2D hyperspectral image of the sample. To reconstruct a high-spatial-resolution image, we first compensated for the image distortion given in the spectrophotometer (Supplementary Fig. 1 and Supplementary Note 1). We then applied an image reconstruction method on the basis of image processing for SIM described in ref. 9 in only the slit direction. Figure 1c shows the spatial frequencies imaged by line illumination (LI) and SLI in the observation of polystyrene beads, showing the resolution improvement in the entire wavelength range by SLI.

### Enhanced spatial resolution and spectral separation capability

**Figure 2:**
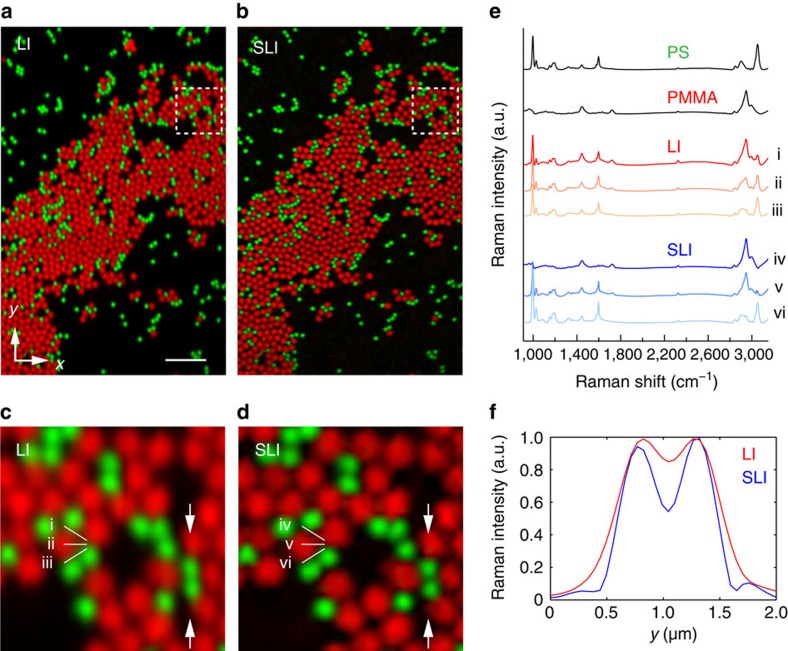
Raman images of polystyrene (green) and poly(methyl methacrylate) (red) beads. (**a**,**b**) LI (**a**) and SLI (**b**) images from the same position in the sample constructed by the Raman signal at 3,055 cm^−1^ (polystyrene, PS) and 2,957 cm^−1^ (poly(methyl methacrylate), PMMA) (Scale bar, 5 μm). (**c**,**d**) The enlarged images of the dotted section in **a** and **b**. (**e**) Raman spectra of PS, PMMA and the spectra measured at the positions indicated by the Roman numerals in **c** and **d**. (**f**) The intensity profiles of the PS beads images indicated by the arrows in **c** and **d** show the clear improvement of the spatial resolution in the SLI mode.

To demonstrate the resulting improvement in spatial resolution by SLI, we observed a mixture of polystyrene (PS) and poly(methyl methacrylate) (PMMA) with diameters of 500 and 800 nm placed on a glass substrate, respectively. The full spectrum is detected for each point, and the intensities of Raman peaks at 3,055 and 2,957 cm^−1^ were used to construct the image contrast of PS and PMMA beads shown in Fig. 2a–d. The Raman spectra obtained in Fig. 2c,d are shown in Fig. 2e. The comparison of the images shows the clear improvement of the image contrast in the SLI mode. The intensity profile of the two PS beads shows that the separation of the neighbouring beads can be clearly performed by the structured illumination (Fig. 2f). The comparison of the Raman spectra in Fig. 2e confirms that the improvement of spatial resolution contributes to avoid the spectral overlap of two materials. For example, in the LI mode, spectra i and iii taken near the edges of the PS and PMMA beads, respectively, contain overlapping spectral features from both PS and PMMA. However, in SLI mode, the spectra corresponding to the same positions (spectra iv and vi) showed no overlap, demonstrating the enhancement of the analytical capability of SLI Raman microscopy. We also confirmed that the resolution improvement is given by the structured illumination and not by the optical transfer function (OTF) compensation used in the reconstruction procedure (Supplementary Fig. 2 and Supplementary Note 2).

### SLI Raman imaging of carbon materials

**Figure 3:**
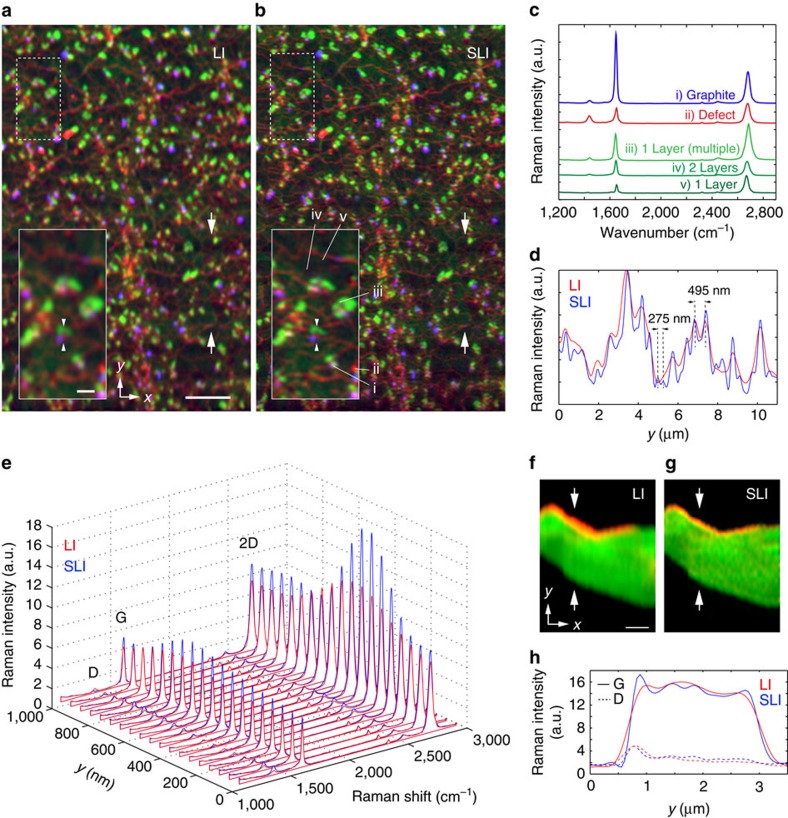
Raman images of carbon materials. (**a**,**b**) LI (**a**) and SLI (**b**) Raman images of a graphene sheet grown by chemical vapour deposition. The images were constructed with the distributions of D (1,307–1,387 cm^−1^ averaged, red), G (peak intensity seen at 1,589–1,598 cm^−1^, blue) and 2D (peak intensity seen at 2,682–2,694 cm^−1^, green) bands (Scale bar, 5 μm). The insets show the enlarged view of the dotted area in the images (Scale bar, 1 μm). (**c**) Raman spectra obtained from the points shown with i–vi in **b**. (**d**) The intensity profile in the distribution of D band indicated by the arrows in **a** and **b**. (**e**) The distribution of Raman spectra obtained from between the arrowheads in the insets in **a** and **b**. (**f**,**g**) LI (**f**) and SLI (**g**) Raman images of a graphite fragment constructed by Raman peaks assigned to D (1,340–1,370 cm^−1^ averaged, red) and G (1,543–1,611 cm^−1^ averaged, green) bands (Scale bar, 1 μm). (**h**) The Raman intensity profiles of the G (solid lines) and D (dash lines) bands between the arrows indicated in **f** and **g**.

In Fig. 3, we show how SLI Raman imaging provides enhanced spatial contrast for chemically distinct features in graphene. Graphene sheets and graphite fragments were observed and compared by the LI and SLI microscope. Nanocarbon-based materials, such as graphene, carbon nanotubes and graphite, are promising research targets owing to their exceptional performance in various applications ranging from the basic science to industrial applications^17^,^18^. As these carbon materials exhibit strong Raman scattering, Raman microscopy is often used to investigate the structural difference in carbon materials^19^. However, many of the features of interest are at scales below the diffraction limit of light, making them difficult to resolve with conventional Raman imaging. In Fig. 3a,b, the intensities of Raman peaks assigned to D (red), G (blue) and 2D (green) vibrational bands are used to visualize the small domain structures. These domains were confirmed as single-layer graphene, two-layer graphene, graphite and defect domains as shown in Fig. 3c. The comparison of Fig. 3a,b shows the unambiguous resolution improvement by SLI, where the reticulate pattern of defects is clearly visualized. The line profiles in Fig. 3d clearly show the improvement of the spatial resolution by SLI. The spectrum distribution shown in Fig. 3e reveals that the D band signal given by SLI has a strong correlation with the G band signal, but not with the 2D band. These correlations cannot be observed in the LI image, indicating the spectral overlap between neighbouring positions owing to the insufficient spatial resolution. We also observed a graphite fragment by the LI and SLI microscope as shown in Fig. 3f,g, respectively. The edge structure of the fragment and fine graphite surface features can be determined from the D and G band distributions only in the SLI imaging modality as shown in Fig. 3h.

### SLI Raman imaging of mouse brain tissue

**Figure 4:**
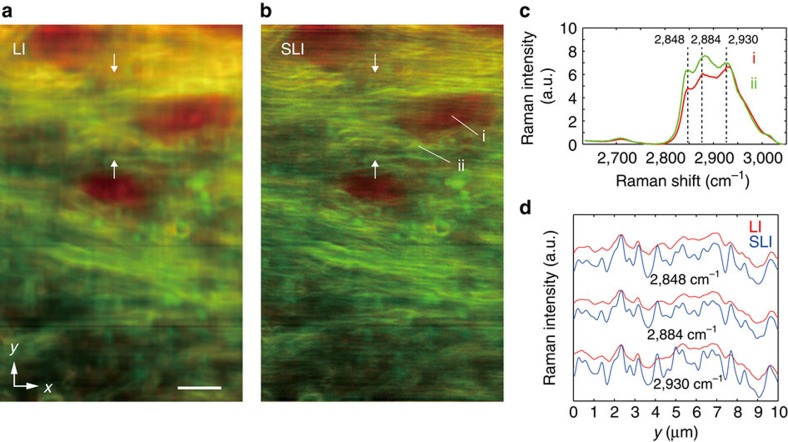
Raman images of a mouse brain slice. (**a**,**b**) The intensity distribution of Raman peaks at 1,682 cm^−1^ (red) and 2,848 cm^−1^ (green) imaged by the LI (**a**) and SLI (**b**) microscopes (Scale bar, 5 μm). Raman peaks at 1,682 cm^−1^ and 2,848 cm^−1^ can be assigned to amide-I and CH_2_ stretching vibrational modes, respectively, predominantly observed in protein beta sheets and lipids. (**c**) Raman spectra measured in **b**. (**d**) The line profiles of three major Raman peaks given in the CH stretching region. The intensity distributions between the two arrows in **a** and **b** are shown.

Biological samples are also emerging as excellent targets of Raman microscopy, as vibrational fingerprints provide information about molecular structures and environments in the sample, which is difficult for conventional labeling techniques to provide^20^,^21^,^22^,^23^,^24^. These samples are typically more complicated than samples used in materials or chemical science, and therefore, the improvement of the spatial resolution is highly beneficial. To demonstrate the applicability of SLI Raman microscopy to biological samples, we observed fixed mouse brain slices, with the resulting reconstructed tissue structures shown in Fig. 4. The images show the distribution of amide-I (1,682 cm^−1^, red) and CH_2_ stretching (2,848 cm^−1^, green) modes typically seen in protein beta sheets and lipids, respectively. Lipid-rich structures in the corpus callosum, which is a bundle of neural fibers with lipid-rich myelin sheaths connecting the two cerebral hemispheres, are clearly resolved only in the SLI image. Figure 4c shows the Raman spectra of CH_2_ and CH_3_ stretching modes in Fig. 4b, which are used for characterizing cellular components^25^, and the spatial line profiles of three typical Raman bands in this spectral region are plotted in Fig. 4d. The three bands obtained with SLI show different spatial distributions. In contrast, the spectra obtained by LI do not present a clear difference in their distributions. Similar spatially resolved images of myelinated neural fibers in mouse brain have been obtained by coherent Raman imaging^26^ using only the CH_2_ symmetric stretch vibration, limiting the analysis to a single band. On the other hand, as full spectral information is readily obtained from the SLI Raman technique, more information from various Raman bands is simultaneously available, making it a more potentially useful tool for diagnosis. These results show that with different chemical components in closer proximity than the resolution capability of standard Raman imaging, the improvement of the spatial resolution also contributes to a more detailed spectroscopic analysis using Raman scattering with spectral discrimination of the distributed components.

## Discussion

In these experiments, the use of structured line illumination in Raman microscopy shows not only spatial resolution improvements but also the increased capability to discriminate component spectra, which is advantageous for the analysis of multi-component samples whose Raman spectra are complicated. For these types of samples, it is common to apply multivariate analysis to extract valuable information from the mixture of Raman scattering. The high spectral discrimination capability of SLI Raman microscopy will help uncover original spectral features more efficiently and reliably from the mixed Raman spectra.

Theoretically, the improvement of spatial resolution is only valid in the direction parallel to the SLI. On the other hand, the spatial resolution in the perpendicular direction is limited by the confocal width of the spectrophotometer. As discussed in ref. 15, SLI microscopy has a trade-off in *x* and *y* spatial resolution because as the structured illumination becomes more closely spaced, the line focusing itself becomes less tightly focused. In our experimental conditions, the normalized offset distance^15^, which is given as the ratio of the spatial frequency of the illumination fringe to the cut-off frequency in the illumination optics, is 0.56 (for the dry objective lens) and 0.48 (for the water immersion objective lens). These illumination conditions offer nearly isotropic spatial resolution in the lateral directions and 1.4-fold improvement of the spatial resolution along the slit direction compared with the theoretical cut-off in wide-field microscopy^15^. Our technique could provide even higher spatial resolutions by further optimization of the spatial frequency of the illumination pattern and signal-to-noise ratio (SNR) of the system so that an aperture ratio >0.56 could be used. To obtain enhanced spatial resolution equally in both *x* and *y* directions, the illumination pattern or sample can be rotated. In such a case, an optical image rotator has to be placed between the sample plane and the edge filter before the spectrometer, to rotate back the image of the illuminated line parallel to the slit.

In point-scanning confocal Raman microscopy, the theoretical limit of spatial resolution is given by using a detection pinhole smaller than 0.5 Airy disc^27^. However, this resolution is not practically used because the Raman signal, which is essentially weak, loses its intensity as the pinhole size decreases. Hence, in many applications, confocal Raman microscopy typically uses a pinhole size larger than one Airy disc, providing a spatial resolution equivalent to that of wide-field microscopy. On the other hand, SLI Raman microscopy circumvents this trade-off between detector aperture size and Raman signal intensity. A smaller slit width can be used without a large loss in the Raman signal intensity because the signal passes through the slit along the parallel direction. The increase in spatial resolution perpendicular to the slit can then be compensated by the gain in spatial resolution along the slit direction through the application of structured illumination. Therefore, SLI Raman microscopy can offer a spatial resolution beyond the practical limit of point-scanning confocal Raman microscopy.

The maximum possible enhancement in spatial resolution can be obtained by using the finest illumination fringe, as implemented in fluorescence SIM. However, the weak signals of Raman scattering restrict the practically resolvable spatial-frequency, making it difficult to separate the high-spatial frequency components and overlay the extracted image components in Fourier space without a gap. The use of slightly wider fringe spacing by SLI ensures the overlap of the extracted image components in Fourier space even in a low-SNR system. This is also the reason why the spatial resolution with SLI can reach nearly double that of its LI counterpart in practical applications, as demonstrated in our experiments. On the other hand, light focusing in one direction in SLI can provide intense excitation intensity, allowing us to detect Raman signals with good SNR.

It is also possible to implement the structured pattern in wide-field illumination instead of line illumination. Wide-field illumination can provide a faster image acquisition compared with SLI. However, to obtain a Raman spectrum with wide-field Raman microscopy^28^,^29^, scanning the detection wavelength or excitation wavelength is required. Using slit-scanning with SLI is more advantageous in hyperspectral Raman imaging because of its capability for much higher spectral resolution although it depends on the number of detected Raman modes.

To optimize the resolution enhancement by SLI in actual experiments, a higher SNR is necessary to obtain the high spatial frequency information more efficiently by the SLI technique. We could increase the SNR by using a longer exposure time. Presently, our system can acquire one SLI image in 1–2 h, primarily owing to the successive acquisition of three images obtained with different illumination phases required for SLI image reconstruction. However, the longer acquisition time could limit the possible applications of SLI spontaneous Raman imaging. For achieving a higher acquisition speed, three different phase images can be obtained simultaneously by utilizing the difference of illumination polarization indicated in ref. 30. SLI Raman microscopy can also implement polarization imaging for non-polarization-dependent samples. We could also utilize multi-colour excitation to obtain different illumination phase images simultaneously^31^. These approaches require very precise knowledge of the polarization or wavelength dependence of the specimen and careful selection of the experimental parameter.

In light of recent advances in coherent Raman imaging using broadband sources^32^, a comparison with the present work is necessary. Coherent anti-Stokes Raman scattering (CARS) can achieve a spatial resolution higher than the linear diffraction limit owing to third-order nonlinear effects. However, the long near-infrared wavelengths normally used in CARS place a restriction in the maximum attainable spatial resolution. On one hand, the present SLI Raman microscopy utilizes structured illumination at a visible wavelength to increase the spatial resolution at levels higher than CARS with capability of further increase, which is limited in practice only by SNR. Although CARS is much faster than spontaneous Raman imaging, the simpler setup, the ability to acquire the entire molecular spectrum, and higher spectral resolution capability of SLI Raman microscopy make it a competitive technique for chemical imaging.

In summary, SLI Raman microscopy shows a well-balanced performance in spatial and spectral resolution with the capability of optical sectioning and powerful spectral analysis. The spatial resolution provided by SLI can reach the theoretical limit of confocal microscopy in many practical applications. Considering the SNR in the detection of Raman scattering, SLI Raman microscopy can realize a remarkable improvement in spatial resolution over that of current Raman imaging techniques, and which can further expand the application of Raman microscopy in various research fields such as material sciences, life sciences and pharmaceuticals. For example, our technique can lead to the detailed analysis of graphene, which is important in understanding fundamental questions on graphene sheet growth mechanism^33^ and characterization of graphene devices^34^. In cell biology, as fluorescence SIM has contributed to visualize the detailed structure of cellular components^35^, it might also be possible to spectrally distinguish these components even without staining^36^ by using the SLI Raman technique. In pharmaceuticals^37^, an emerging demand that can benefit from the high spatial resolution of SLI Raman microscopy is the precise determination of the distribution of the different chemical components in a drug.

## Methods

### Experimental set-up

A CW frequency-doubled Nd:YVO_4_ laser (532 nm, Spectra-Physics, Millenia) was used as a light source for all the experiments. An imaging spectrophotometer (Bunko Keiki, MK-300) equipped with a cooled CCD camera (Princeton Instruments, PIXIS400B) was used for detecting Raman spectra. A total of 400 Raman spectra were measured simultaneously in one exposure. Two edge filters (Semrock, LPD01-532RU-25) were used to separate the laser and the Raman scattering light before the spectrophotometer. A commercial inverted microscope (Nikon, ECLIPSE Ti) was used for mounting the objective lens and sample, and a focus stabilization system (Nikon, Perfect Focus System) was used to keep the focal plane in the sample constant during the measurement. In all Raman imaging experiments, the slit width was set to the Airy size with the centre wavelength (591 nm) detected in the spectrophotometer, which corresponds to a spectral resolution of 3.1 cm^−1^ at the centre wavelength.

### Image processing

First, we removed the cosmic rays detected in the measured spectra by using a median filter on the recorded CCD frame. The median filter was applied to a 5 × 5 pixels area centred on the pixel exhibiting a cosmic ray spike, which shows as a large spurious intensity far higher than the typical Raman intensity. The process was skipped if the cosmic ray did not exceed a threshold (typically six standard deviations above the local median). Cosmic ray spikes occur only at a few pixels in the CCD array, which means that the vast majority of the raw Raman data is unaffected by the median filtering process. The spectral data were then processed by subtraction of the CCD read-out background and pixel resampling for distortion compensation (Supplementary Fig. 1 and Supplementary Note 1). For SLI images, we used the SIM reconstruction procedure described in ref. 9. Briefly, we extract the spatial frequency components in the Fourier domain, and combine them appropriately with OTF compensation after moving the components back to their original positions in Fourier space. For OTF compensation, a Wiener filter is used with a parameter that is empirically determined as described in the SIM literature (see for example: refs 9, 38) to minimize artifacts that arise during the image reconstruction process. The OTF of the imaging optics was determined from an image of a fluorescent bead with a diameter of 40 nm. In addition, a triangle window function is used to suppress edge-related artifacts. The reassembled image is then transferred to the real space domain. For LI images, we averaged three Raman images obtained with different fringe phases and resampled the resultant LI image by using spline interpolation to make the pixel number equal to that in the reconstructed SLI image. We applied singular value deposition (SVD) to the PS/PMMA image and the brain tissue image for noise reduction^39^. Briefly, SVD models the data into two sets of vectors weighted by singular values and by approximating the data matrix as a subset of singular values and vectors that contains primarily the relevant spectral features, the noise present in the data is reduced. Visual inspection of the rejected SVD vectors was performed to ensure that they contain predominantly noise and no strong spatial and spectral features. Savitzky–Golay fitting was applied to the graphene spectrum for smoothing. The fluorescence background was removed using a polynomial curve fitting technique^40^ in the PS/PMMA beads and the brain tissue images.

### Imaging of PS and PMMA mixture

PS and PMMA particles were dispersed in water and dropped onto a glass substrate (Matsunami, MAS-coated glass). An objective lens with an NA 1.27 WI, × 60 (Nikon, CFI Plan Apo IR × 60) was used for the observation. A phase grating of 92 grooves per millimetre (GPM), corresponding to a normalized offset distance of 0.48 (ref. 15), to produce the interference fringe on the line illumination, was used. The exposure time and excitation intensity were 5 s per line and 4.3 mW μm^−2^, respectively. The pixel numbers of the image in the measurement were 200, 400 and 900 for *x*, *y* and *λ* directions, respectively. The scanning step size for the *x* direction was 143 nm.

### Graphene imaging

A CVD graphene sheet was purchased from Graphene Platform and placed on the microscope stage for observation. An NA 0.95 dry objective lens (Nikon, CFI Plan Apo λ × 60) was used for the observation. A phase grating of 80 GPM, corresponding to a normalized offset distance of 0.56 (ref. 15), to produce the interference fringes in the line illumination, was used. The exposure time and the excitation intensity were 10 s per line and 2 mW μm^−2^, respectively. The pixel numbers of the image in the measurement were 200, 400 and 900 for *x*, *y* and *λ* directions, respectively. The scanning step size for the *x* direction was 143 nm.

### Graphite imaging

Thin layered graphite was prepared through mechanical cleavage of highly ordered pyrolytic graphite (HOPG; SPI supplies, 476HP-AB). The same objective lens and the phase grating pair used in the PS/PMMA imaging were applied. The exposure time and the excitation intensity were 5 s per line and 0.85 mW μm^−2^, respectively. The pixel numbers of the image in the measurement were 50, 400 and 900 for *x*, *y* and *λ* directions, respectively. The scanning step size for *x* direction was 109 nm.

### Mouse brain tissue imaging

Animal experiments were performed in accordance with the guidelines of the Japanese Pharmacological Society, and were approved by the Animal Care and Use Committee of the Graduate School of Pharmaceutical Sciences, Osaka University. All efforts were made to minimize the number of animals used. Adult male C57BL/6 mice were anaesthetized with sodium pentobarbital (50 mg kg^−1^, intraperitoneally), and perfused through the left ventricle with 4% paraformaldehyde in phosphate-buffered saline (4% PFA). Brains were removed, fixed overnight at 4 °C in 4% PFA and sliced at a thickness of 20 μm in phosphate-buffered saline on a Leica vibratome (VT 1000S). The same objective lens and phase grating pair used in the PS/PMMA imaging were applied. The exposure time and the excitation intensity were 10 s per line and 4.3 mW μm^−2^, respectively. The pixel numbers of the image in the measurement were 200, 400 and 900 for x, y and λ directions, respectively. The scanning step size for the *x* direction was 143 nm.

## Additional information

**How to cite this article:** Watanabe, K. *et al.* Structured line illumination Raman microscopy. *Nat. Commun.* 6:10095 doi: 10.1038/ncomms10095 (2015).

## Supplementary Material

Supplementary Information Supplementary Figures 1-2 and Supplementary Notes 1-2
