# Supplementary material for: Structured line illumination Raman microscopy
Source: Nat Commun. 2015 Dec 2;6:10095. doi: 10.1038/ncomms10095 (PMC4686755; doi:10.1038/ncomms10095)
Supplement: Supplementary Information — Supplementary Figures 1-2 and Supplementary Notes 1-2 [file ncomms10095-s1.pdf]

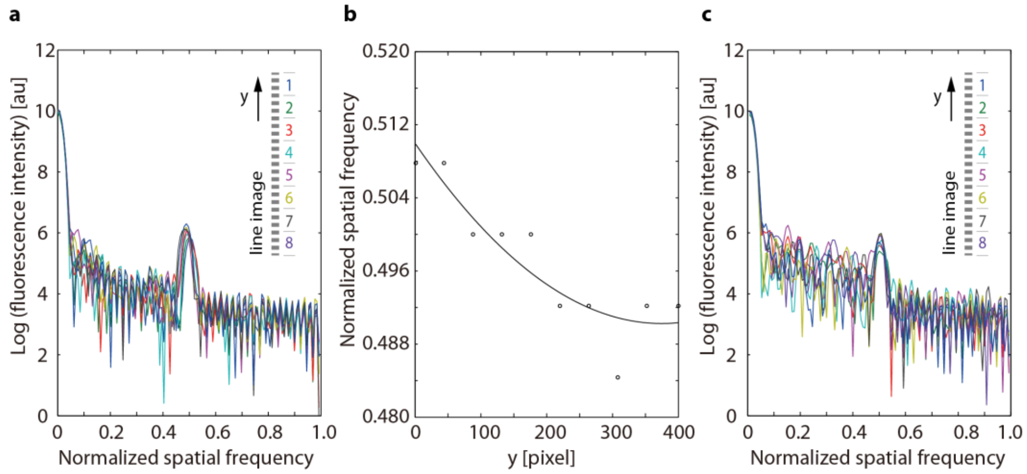

Supplementary Figure 1: Compensating for the distortion in the line image. a, **b**, Fourier spectra (**a**) and the peak frequencies (**b**) of the interference fringes at different positions in a SLI. **c**, Fourier spectra after distortion compensation of **a**.

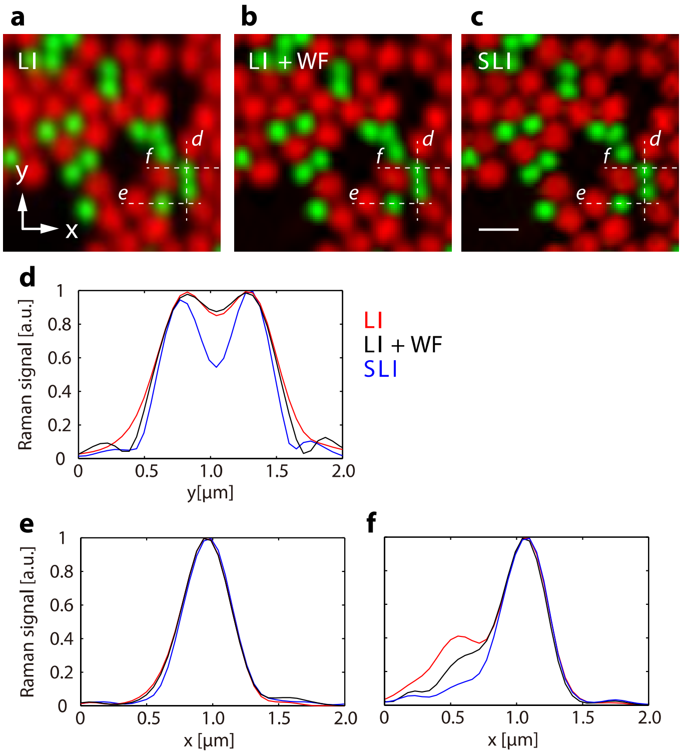

Supplementary Figure 2: Comparison of the PS/PMMA beads images. a-c, PS (green) /PMMA (red) beads images obtained by LI (**a**), LI with Wiener filtering (**b**) and SLI (**c**) (Scale bar, 1 μm). **d-f**, The line profiles across the PS beads shown by the dashed lines in the images.

Supplementary Note 1: Compensating for the distortion in the line image

Due to the aberration generated by the imaging optics in a Czerny-Turner spectrophotometer, the hyperspectral line image has distortion along the x axis, which prevents performing SIM reconstruction appropriately in the entire image.

To compensate for the distortion, we first measured the amount of the distortion by obtaining a line image of a fluorescent film by the SLI microscope and applying Fourier transform to the segments of the line image. In our experiments, a line image was segmented into 8 segments to determine the position dependent spectra as shown in Supplementary Fig. 1a. We then calculated the position dependent fringe frequency and applied curve fitting to determine the amount of image distortion (Supplementary Fig. 1b). Based on this curve fitting result, we resampled the image data along the x axis to compensate the distortion. Supplementary Fig. 1c shows the Fourier spectra from each segment after the distortion compensation, where the peaks of the spectra are well matched.

Supplementary Note 2: Comparison of the PS/PMMA beads images

To confirm that the resolution improvement is given by structured line illumination (SLI), we compared the following three images of a mixture of PS and PMMA beads obtained by LI, LI image with Wiener filtering (WF) and SLI as shown in Supplementary Figure 2a-c. These images show the same area displayed in Fig. 2c,d. In the LI + WF image, Wiener filtering was applied only along the y-axis, which is the same axis as the structured line illumination pattern used in the SLI image. The same Wiener filter parameter was used for the LI + WF image and the SLI image. Supplementary Figs. 2d-f show the line profiles across the PS beads indicated by the dashed lines in Supplementary Fig. 2a-c. Along the y-axis (Supplementary Fig. 2d), comparison of the line profiles confirm that the spatial resolution in the SLI image is higher than in the LI+WF image, which in turn shows only a slight improvement compared to the LI image. Along the x-axis, we compared the line profiles across an isolated PS bead (no adjacent PS beads, Supplementary Fig. 2e) and one with adjacent PS beads (Supplementary Fig. 2f). In both cases, comparison of the line profiles clearly shows no resolution improvement in the x-axis. In Supplementary Fig. 2f, the line

profile of the bead shows a considerable overlap with that of an adjacent bead in the LI image but this overlap is significantly reduced in the SLI image. This decrease in overlap between adjacent beads seemingly produces a better separation in the x-axis in the SLI image because the resolution improvement in the y-axis due to SLI reduces the blur between adjacent particles not only in the y-axis but in the x-axis as well.

The above results prove that the resolution improvement in SLI Raman microscopy is given by the effect of SLI and not by the OTF compensation. The resolution improvement is observed only in the axis where the structured illumination pattern varies.
